# Development of Polydopamine Forward Osmosis Membranes with Low Reverse Salt Flux

**DOI:** 10.3390/membranes10050094

**Published:** 2020-05-10

**Authors:** Pelin Oymaci, Kitty Nijmeijer, Zandrie Borneman

**Affiliations:** Membrane Materials and Processes, Department of Chemical Engineering and Chemistry, Eindhoven University of Technology, P.O. Box 513, 5600 MB Eindhoven, The Netherlands; p.oymaci.akin@tue.nl (P.O.); d.c.nijmeijer@tue.nl (K.N.)

**Keywords:** forward osmosis, polydopamine, temperature, surface modification, polyethersulfone

## Abstract

Application of forward osmosis (FO) is limited due to membrane fouling and, most importantly, high reverse salt fluxes that deteriorate the concentrated product. Polydopamine (PDA) is a widely used, easily applicable, hydrophilic, adhesive antifouling coating. Among the coating parameters, surprisingly, the effect of PDA coating temperature on the membrane properties has not been well studied. Polyethersulfone (PES) 30 kDa ultrafiltration membranes were PDA-coated with varying dopamine concentrations (0.5–3 g/L) and coating temperatures (4–55 °C). The quality of the applied coating has been determined by surface properties, water permeability and reverse salt flux using a 1.2 M MgSO_4_ draw solution. The coating thickness increased both with the dopamine concentration and coating temperature, the latter having a remarkably stronger effect resulting in a higher PDA deposition speed and smaller PDA aggregates. In dead-end stirred cell, the membranes coated at 55 °C with 2.0 g/L dopamine showed NaCl and MgSO_4_ retentions of 41% and 93%, respectively. In crossflow FO, a low reverse MgSO_4_ flux (0.34 g/m^2^·h) was found making a very low specific reverse salt flux (J_s_/J_w_) of 0.08 g/L, which outperformed the commercial CTA FO membranes, showing the strong benefit of high temperature PDA-coated PES membranes to assure high quality products.

## 1. Introduction

**Figure 1:**
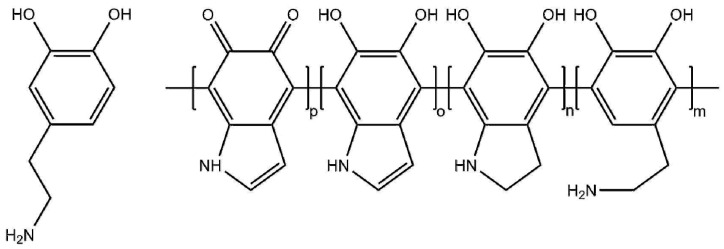
Proposed structure of a polydopamine chain consisting of covalently linked cyclized units containing indole, catechol, quinone, and amine groups [17,18].

Forward osmosis (FO) is an emerging membrane process that uses only the osmotic pressure difference between two solutions as the driving force for water transport. Application of FO is advantageous to concentrate dilute, shear, and pressure-sensitive rest streams energy efficiently in the presence of a high concentration draw solution [1,2]. There is an increase in the number of studies related to FO that investigate and improve the process conditions as well as FO membrane properties to enhance the efficiency of the process [3]. However, industrial application of FO is still limited due to poor performance of the existing membranes that suffer from high reverse salt flux (J_s_) and low water flux (J_w_), internal concentration polarization (ICP) and fouling [3,4,5,6]. In addition, energy efficient regeneration of the draw solution is an important bottleneck still [7] and has brought up the research on hybrid systems with FO such as FO-membrane distillation [8], FO-reverse osmosis (RO) [9], and FO-nanofiltration (NF) [10]. The latter hybrid system shows promising results that can recover draw solutions energy efficiently using draw solutes based on divalent ions such as Na_2_SO_4_ or MgSO_4_ due to the higher rejection rates [11,12], allowing the use of NF instead of tight RO membranes [13,14]. Based on these important criteria, the most critical factors for successful FO operation are a low J_s_ and a high J_w_. In that case, product quality is not affected by the reverse salt flux and loss of driving force is prevented [2]. In addition to this, the reverse salt flux is also found to play an important role in terms of fouling of FO membranes [15]. The ratio of J_s_ and J_w_ is characterized as the specific reverse salt flux and effectively decreasing its value is the main challenge in FO membrane development. Surface coating is one of the most effective methods for membrane modification to obtain low reverse salt fluxes, high water fluxes, hydrophilicity, and antifouling properties [16]. Dopamine and polydopamine (PDA) (Figure 1) have gained great attention as thin surface coatings due to material-independent surface adhesion and antifouling properties.

Dopamine polymerizes spontaneously in alkaline conditions and adheres onto a wide variety of surfaces with a single immersion step [19]. Despite its wide application, the exact polymerization mechanism is still under debate with evidences for both covalent and non-covalent bonding [20]. Delparastan et al. used single-molecule force spectroscopy to investigate the covalent and non-covalent bonding of a PDA film [18]. They found that PDA films contain high molecular weight polymer chains with covalently bound subunits and reversible intramolecular interaction-based non-covalent bonds. It was also found that PDA film formation starts at the solid–liquid interface with adsorbed oligomers that polymerize further and form high molecular weight chains. Their findings are in agreement with the covalent PDA model proposed by Liebscher et al. [17] that PDA chains consist of covalently linked oxidized and cyclized units containing indole, quinone, catechol, and amine functional groups (Figure 1). PDA is applied in many membrane processes as an effective antifouling surface as a primer that allows immobilization of functional groups [21,22,23] or as a selective separation layer [24]. PDA is investigated as a separation layer in several studies by depositing the layer together with other components or as self-polymerized layer. Cheng et al. [25] coated polyethersulfone (PES) UF membranes with PDA to form a biomimetic coating and studied the effect of the coating time, initial dopamine concentration and solution pH on surface properties, permeability, BSA ultrafiltration (UF) performance, platelet adhesion, and blood coagulation. It was found that PDA improved the hydrophilicity and the antifouling properties of the membranes. Lv et al. co-deposited PDA with polyethyleneimine to form nanofiltration membranes on hydrolyzed polyacrylonitrile ultrafiltration membranes and investigated the permeability, rejection, and hydrophilicity of the membranes [26]

In a few studies, the coating temperature was found to have an important influence on the reaction kinetics, resulting in finer PDA aggregates and an increase in thickness by accelerating the PDA deposition [27,28]. However, the effect of coating temperature in relation to FO membrane performance was not studied systematically although it is an important parameter that affects the PDA coating properties. Here, we show how the PDA coating temperature can be used as an effective parameter to control the membrane properties and with that to steer J_s_ and J_w_ and thus the FO membrane performance. To the best of the authors’ knowledge, this study is the first to investigate the effects of the coating temperature on the separation performance of a PDA-coated PES UF membrane support. The effect of coating temperature and dopamine concentration on the FO performance of the membranes is evaluated together with a series of membrane characterization techniques to obtain information on its morphology, surface chemistry, hydrophilicity, and surface charge. 

## 2. Materials and Methods

### 2.1. Materials

**Figure 2:**
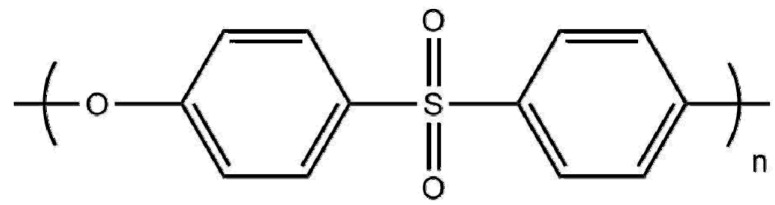
Molecular structure of polyethersulfone.

Polyethersulfone (PES; Figure 2) flat-sheet ultrafiltration membranes (MWCO 30 kDa) were kindly provided by Sartorius AG (Göttingen, Germany).

Ultrapure water (UPW) was obtained from an ELGA Purelab (VWS, High Wycombe, UK) water purification system (18.2 MΩ·cm, 1.2 ppb TOC) and used to prepare all solutions. Dopamine hydrochloride, tris(hydroxymethyl)aminomethane (Tris), potassium chloride, and potassium hydroxide were purchased from Sigma-Aldrich (Merck, Darmstadt, Germany). Magnesium sulfate heptahydrate, ethanol (absolute), and acetone were purchased from VWR Chemicals (Radnor, Pennsylvania, USA). Sodium chloride (Sanal^®^ P) was kindly supplied by Nouryon (Amsterdam, The Netherlands), and 1 M hydrochloride solution was purchased from Merck (Darmstadt, Germany). All chemicals were used as received.

### 2.2. Polydopamine Coating

PES membranes were immersed in 18 vol% ethanol/UPW solution overnight and then rinsed with UPW. After rinsing, the membranes were stored in fresh UPW at 4 °C until use. PES membranes were mounted in a custom-made mold (active area ~150 cm^2^) to make sure that only the selective layer of the membrane was exposed to the coating solution. The mold was immersed in the dopamine coating solution (100 mL for 130–140 cm^2^ surface area) with varying initial dopamine concentrations (0.5, 2.0, and 3.0 g/L) and coating temperatures (4, 25, and 55 °C). The dopamine coating solution was prepared by dissolving dopamine in a Tris solution (10 mM, pH 8.5). The solution was shaken at the desired temperature at 50 rpm for 24 h. Always, freshly prepared coating solutions were used. The coated membranes were sonicated for 30 min in UPW to remove weakly bound PDA precipitates and kept in UPW until further use.

### 2.3. Membrane Characterization

#### 2.3.1. Field Emission Scanning Electron Microscopy (FESEM)

Surface and cross section morphologies of the gold or platinum sputter coated membranes after vacuum drying were characterized by field emission scanning electron microscopy (Quanta 3D FEG, FEI, Waltham, MA, USA), operating at an acceleration voltage of 5 kV. Cross section samples were fractured in liquid nitrogen before sputter coating.

#### 2.3.2. X-Ray Photoelectron Spectroscopy

Surface chemistry of the vacuum dried membranes was revealed by X-ray photoelectron spectroscopy (XPS; Thermo Scientific K-Alpha, Waltham, MA, USA). The system was equipped with a monochromatic small-spot X-ray source and a 180° double focusing hemispherical analyzer with a 128-channel detector. Spectra were obtained using an aluminum anode (Al Kα = 1486.6 eV) operating at 72 W and a spot size of 400 µm. Survey scans with a penetration depth of approximately 10 nm were measured at a constant pass energy of 200 eV and region scans were obtained at 50 eV. The background pressure was 2 × 10^−9^ mbar and during the measurement 3 × 10^−7^ mbar argon was used because of the charge compensation dual beam source.

The depth profiling method was used to observe the composition profile of the membranes at different depths. Ion sputtering with 3000 eV energy in the etching mode with an estimated Ta_2_O_5_ sputter rate of 0.25 nm/s was used. The size of the measured area was 400 μm.

#### 2.3.3. Contact Angle Measurement

Surface hydrophilicity was characterized using a sessile drop method with a contact angle goniometer type (DataPhysics, OCA, Filderstadt, Germany) at ambient temperature. UPW droplets of 2 µL were used to measure the contact angle of three different dried membrane samples on five different areas. Average values and standard deviations were calculated using both the right and left contact angles.

#### 2.3.4. Zeta Potential Measurement

The surface charge of the unmodified and modified dry membranes was determined using an electrokinetic analyzer (SurPASS™ 3, Anton Paar, Graz, Austria) equipped with an adjustable gap cell (1 cm × 2 cm). One millimolar potassium chloride background solution was used over a pH range of 2.5 to 9.5. The electrolyte solution was initially adjusted to pH 9.5 with a 50 mM KOH solution and then decreased stepwise automatically by the instrument to pH 2.5 with 50 mM HCl solution. The zeta potential was then calculated based on measured streaming current values.

#### 2.3.5. Membrane Performance Evaluation

##### Dead-End Membrane Filtration

The permeability and rejection of the coated membranes were determined using “Amicon type” collected dead-end stirred cells. The water flux (J_w_, L/m^2^·h) was calculated by dividing the permeate volume by the time elapsed and the membrane area (39 cm^2^). The membranes were first compacted at 5 bar, using N_2_ as propellant, for 3 h until a steady flux was reached. Then, the UPW permeability (*A*, L/m^2^·h·bar) was determined from the slope of a flux-pressure plot ranging from 1 to 5 bar with a step size of 1 bar (Equation (1)):

(1) $$A=\frac{J_{w\;}}{\Delta P}$$

where ΔP is the pressure difference (bar). Aqueous NaCl and MgSO_4_ salt solutions (2 g/L) were used for the rejection measurements at 5 bar. Rejection (R, %) was calculated with Equation (2), where c_f_ and c_p_ are the feed and permeate concentration (g/L), respectively.

(2) $$R=\left(\frac{c_{f}\;{-\;c}_{p}}{c_{f}}\right)\;\times \;100$$

##### Forward Osmosis Test

Forward Osmosis (FO) filtration measurements were conducted on a cross-flow FO filtration system (Convergence Industry B.V., Enschede, The Netherlands). The membrane holder has a flow channel on both sides of the membrane with an effective filtration area of 0.006 m^2^ (40 mm width, 150 mm length) and 5 mm slit height. The membrane was mounted with the active layer facing the feed solution (FO mode). Two diamond-shaped spacers with a thickness of 2 mm were used on both sides of the membrane. UPW was used as feed solution (1.8 L) and 1 L MgSO_4_ solution was used as a draw solution with an initial concentration of 1.2 M. The co-current flow rates were set to 36 L/h (25 cm/s). Measurements were performed at ambient temperature and continued until 250 mL water was permeated from the feed to the draw solution side [29]. The water flux J_w_ (L/m^2^·h) was calculated from the mass increase in the draw solution compartment that was converted to permeate volume V_d_ (L) in a certain time t (h) per membrane area A (0.006 m^2^) (Equation (3)). The change in feed side conductivity in time was used to calculate the reverse salt flux J_s_ (g/m^2^·h) from the change in salt concentration c (g/L), the feed solution volume V_f_, the time elapsed, and the membrane area (Equation (4)). Specific reverse salt flux was calculated as the ratio of the reverse salt to water flux J_s_/J_w_ (g/L).

(3) $$J_{w}=\frac{V_{d}}{A\;\cdot \;t}$$

(4) $$J_{s}=\frac{c\;\cdot {\;V}_{f}}{A\;\cdot \;t}$$

## 3. Results and Discussion

### 3.1. Surface Morphology by FESEM

#### 3.1.1. Effect of Dopamine Concentration

**Figure 3:**
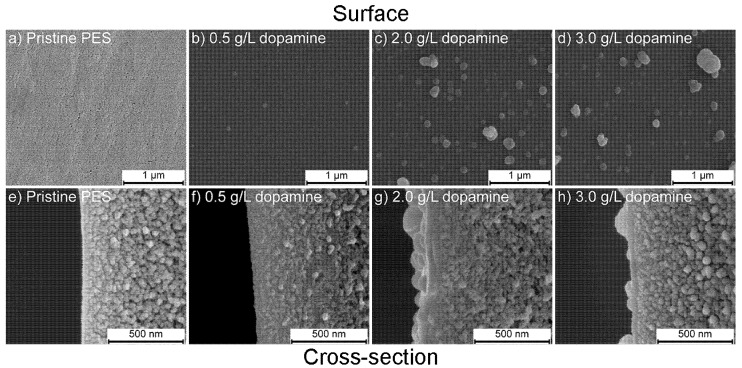
Field emission scanning electron microscopy (FESEM) images that show the effect of the dopamine concentration on the coating morphology at 25 °C. Surface and cross section images of (**a**,**e**) pristine PES support; (**b**,**f**) 0.5 g/L; (**c**,**g**) 2.0 g/L; (**d**,**h**) 3.0 g/L at a magnification of 100,000× (scale bar: 1 μm) and 250,000× (scale bar: 500 nm), respectively.

Figure 3 shows the FESEM images of the surface and cross-section of the membranes coated with different dopamine concentrations at 25 °C.

Figure 3a,e shows the surface and cross section of the pristine PES 30 kDa membrane. The pristine membrane shows a smooth surface with a pore radius in the order of 5 nm [30]. The pore size and porosity gradually decrease from bottom to top of the membrane. Figure 3b–d,f–h illustrates the surface and cross section after 24 h coating with, respectively, 0.5, 2.0, and 3.0 g/L dopamine solutions. The membrane surfaces are well covered with the PDA coating and pores are no longer visible. The images clearly show that the PDA is deposited in the form of aggregates that vary in number and size. When coatings using a 0.5 g/L solution are applied, the coating layer contains only a few small aggregates. At higher coating concentrations, more and larger PDA aggregates appear on the surface without any visible difference between 2.0 and 3.0 g/L. Formation of PDA aggregates and its dependence on the dopamine concentration were studied by Vecchia et al. [31] using dynamic light scattering. They found that PDA starts forming small oligomers from the beginning of the polymerization. These PDA oligomers act as seeds for the growth of bigger aggregates. The hydrodynamic diameter of the PDA aggregates increase with the initial dopamine concentration of the coating solution. The observed differences in aggregate size also affect the thickness and roughness of the obtained coatings, as seen from the cross section images (Figure 3f–h). The coating layer obtained with a 0.5 g/L dopamine solution is thin and not clearly visible on the cross-sectional image. The roughness and coating layer thickness increase with the dopamine concentration in the coating solution due to the formation and deposition of more and larger aggregates and this layer is clearly visible on the 2.0 and 3.0 g/L dopamine coated membranes. Kasemset et al. [32] investigated the PDA coating thickness on polysulfone ultrafiltration membranes using ellipsometry and found only a slight thickness difference when comparing coatings formed from 2 or 4 g/L dopamine concentration. Moreover, here differences between 2.0 and 3.0 g/L dopamine coated membranes are small.

#### 3.1.2. Effect of Coating Temperature

**Figure 4:**
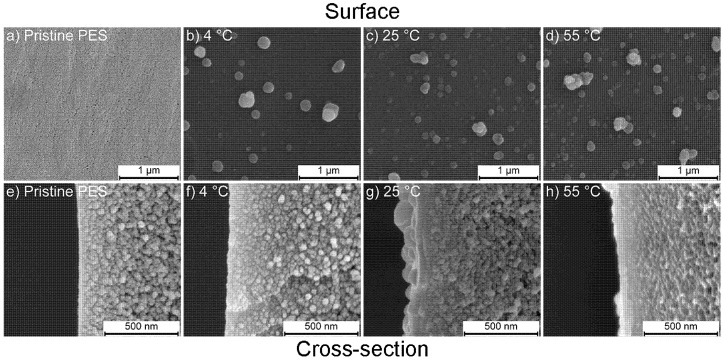
FESEM images that show the effect of coating temperature on the morphology of the membranes coated with 2.0 g/L dopamine. Surface and cross section images of (**a**,**e**) pristine PES support; (**b**,**f**) 4 °C; (**c**,**g**) 25 °C; (**d**,**h**) 55 °C at a magnification of 100,000× (scale bar: 1 μm) and 250,000× (scale bar: 500 nm), respectively. Figure 3a,c,e,g was added to make comparison easier.

The effect of temperature on the coating morphology after 24 h of coating is depicted in the FESEM images in Figure 4.

Figure 4b–d,f–h shows the surface and cross section images of the temperature dependent coatings using the 2.0 g/L dopamine coating solution. The FESEM images clearly demonstrate the increasing amount of deposited PDA aggregates embedded in the coating layer with increasing coating temperature. Membranes coated at 4 °C show only a few PDA aggregates, varying in dimensions. Membranes coated at higher temperatures contain more but smaller PDA aggregates. This is attributed to the faster polymerization reaction of PDA at higher temperatures as also found by Zhou et al. who studied the effect of the coating temperature on the PDA polymerization kinetics using a quartz-crystal microbalance (QCM). They found that a higher reaction temperature promotes the oxidation during PDA polymerization and accelerates the formation of PDA aggregates and therefore increases the amount of PDA mass deposited on the QCM chips [27]. Smaller PDA particle sizes at elevated temperatures were also found in the supernatant of the 2 g/L PDA coating solution after 24 h by Jiang et al. [28] who found that the smaller particle size is due to the acceleration of the polymerization reaction at higher temperature (60 °C). Jiang et al. studied the effect of coating temperature (20–45 °C) on PDA deposition on various hydrophobic supports. It was found that elevated temperatures resulted in thicker PDA layer and rougher surface at the same time due to the higher reaction rate. Figure 4f indeed shows that the PDA layer coated at 4 °C is very thin and hardly distinguishable from the PES support, whereas Figure 4g,h illustrates that increasing the temperatures, clearly results in thicker coating layers.

### 3.2. Surface Chemistry

XPS reveals the surface chemistry of the pristine and PDA-coated PES membranes. Atomic compositions of the surface and nitrogen to carbon and nitrogen to sulfur ratios for the investigated membranes are given in Table 1.

Nitrogen and sulfur percentages are the most important indicators to observe the change of the PES membrane surface upon PDA coating, as sulfur is characteristic for PES and nitrogen has that role in PDA. After PDA coating, the nitrogen atomic percentage of all surfaces increases as a result of PDA deposition. Further, the percentage of sulfur decreases drastically because the dopamine coating covers the sulfur signal from the support due to the limited penetration depth of XPS. In addition, the nitrogen to carbon ratio (N/C) of the PDA-coated membranes is similar to the theoretical ratio of dopamine (0.125) [25], which suggests that a dense coating layer of at least 10 nm, being the surface depth of the XPS measurement, is deposited. The surface coverage of PDA is also well observed in FESEM images (Figure 3 and Figure 4). Even at low dopamine concentration, the pores on the PES pristine membrane are no longer visible due to the well covered surface which is in agreement with XPS results. N/S ratios of the membranes that were coated with 2.0 g/L dopamine show a significantly higher N/S ratio with increasing temperature compared to 4 °C, indicating the presence of higher amounts of nitrogen due to the thicker layer and higher amount of PDA. The same trend is also observed for the membranes coated at higher dopamine concentrations. On the other hand, a decrease in N/S ratio was observed with the increase of the initial dopamine concentration from 2.0 to 3.0 g/L. This ratio is magnified by the very low sulfur content and is in all cases around two orders of magnitude. Despite this, the small decrease in N/S ratio is attributed to the bulkier PDA aggregate formation at higher concentration which may result in a more open PDA layer structure. The nitrogen peak in pristine PES is unexpected and most likely derived from the pore forming agent polyvinylpyrrolidone (PVP), a widely used additive during the production of porous PES membranes. XPS depth profiling was used to reveal coating thicknesses. It gives the composition of the surface after a number of etching steps using ion sputtering with an etching rate of 0.25 nm/s. The sulfur signal was used to quantify the thickness of the PDA layer. This sulfur signal remains constant when the full PDA layer is etched away from the PES surface. 

**Figure 5:**
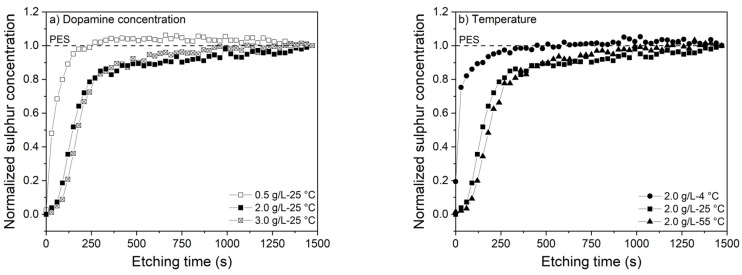
Normalized sulfur profiles of the membranes coated with different (**a**) dopamine concentrations at 25 °C (0.5, 2.0, and 3.0 g/L) and (**b**) temperatures (4, 25, and 55 °C).

Figure 5 shows the sulfur percentage of the membranes normalized by the final sulfur percentage (sulfur content of the pristine PES support) as function of the etching time.

Membranes coated with 0.5 g/L dopamine immediately show a sharp increase in amount of sulfur due to the presence of a several nanometers of PDA coating layer only. The membranes coated with higher dopamine concentrations show a delayed increase due to the thicker layer (~65 nm). The layer thickness of the 2.0 and 3.0 g/L coated membranes is found to be equal also with XPS and the curves mostly coincide. Similar trends are observed for coatings at different temperatures. Coating at 4 °C shows an instantaneous increase from the onset in sulfur percentage, as a result of the thinner layer due to slower PDA deposition. On the other hand, coating at higher temperatures resulted in a more gradual increase due to the formation of thicker PDA layers. Membranes coated at 55 °C have slightly thicker PDA layer than membranes coated at 25 °C. The XPS results are in good agreement with the FESEM observations, showing similar thicknesses of PDA layers approximated by using the etching rate of 0.25 nm/s.

### 3.3. Surface Hydrophilicity

The sessile drop method reveals the effect of the dopamine concentration and temperature on the contact angle of the membranes. The pristine PES support and the support coated with PDA at 25 °C results in similar contact angles, independent of the coating concentration (58° ± 8°). Considering the fact that surface hydrophilicity is affected by both surface chemistry and surface roughness, this is partially because of the addition of hydrophilic PVP as additive to PES upon membrane preparation, although largely washed out as shown by the XPS measurements. Another reason is the PES membrane surface porosity which is found to lower the contact angle of the intrinsically hydrophobic PES membrane [33]. The obtained contact angle results are also in agreement with the contact angle measured by Cheng et al. [25] who found a value of 55.8° without a significant change when the dopamine concentration was increased. Membranes coated with PDA at 4 °C show similar contact angle values (59° ± 4°) as pristine PES support and PDA-coated membranes at 25 °C, although the roughness seems to increase in the FESEM images as shown in Figure 4. However, a significant decrease in the contact angle is observed for membranes coated at 55 °C (36° ± 9°). As the surface chemistry is the same, this result can only be attributed to a significantly increased surface roughness of the coating layer. This is supported by the FESEM images: At 55 °C, an increase in the amount of PDA deposited is visible in the form of more smaller and bigger aggregates changing the roughness of the surface and thus the contact angle. The Wenzel model states that for a chemically homogeneous surface, roughness promotes the surface hydrophilicity by reducing the apparent contact angle [34,35,36]. Cheng et al. [25] showed that the roughness of the surface increased with the coating time resulting in a lower contact angle. Consequently, the decrease in apparent contact angle observed at 55 °C stems from an increase in surface roughness.

### 3.4. Surface Charge

**Figure 6:**
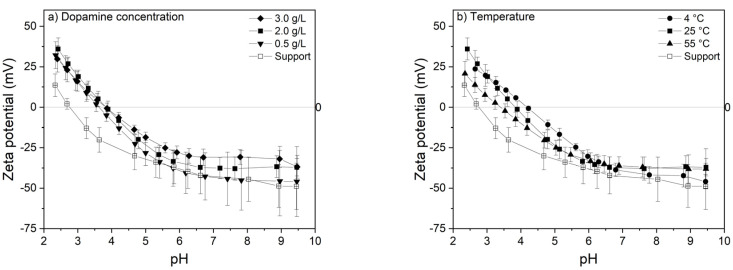
Effect of (**a**) dopamine concentration (temperature: 25 °C) and (**b**) temperature (dopamine concentration: 2.0 g/L) on surface zeta potential of the investigated membranes. Electrolyte: 1 mM aqueous KCl solution. Average values and standard deviations represent three replicate samples.

Effect of the dopamine concentration and temperature on the surface charge of the investigated membranes can be seen in Figure 6.

PES membranes have a negative charge above their isoelectric point at pH 3.0. This is explained by the adsorption of hydroxide ions from the solution to the uncharged, hydrophobic surface of the membrane, which is measured as negative charge [37]. Coating of PES membranes with PDA results in a shift in the isoelectric point to higher pH values, confirming the deposition of more basic entities on the surface. Due to the abundant presence of phenolic hydroxyl groups in the PDA structure, the isoelectric point is shifted towards more basic pH giving negative zeta potential values above pH 4 for coated membranes because OH dissociates in water and remains negatively charged after it donates H^+^ [37]. Below this pH, the amine functional groups are protonated and the surface charge becomes positive [38,39]. Zeta potential measurements at acidic and basic pH did not show a significant change with varying dopamine concentration nor temperature as a result of similar functional groups present in the coating. Notwithstanding, also here the 0.5 g/L and the 4 °C-based coating, the thinnest coatings, are the closed to the pristine PES membrane.

### 3.5. Membrane Performance

#### 3.5.1. Dead-End Filtration Performance

##### Effect of Dopamine Concentration

**Figure 7:**
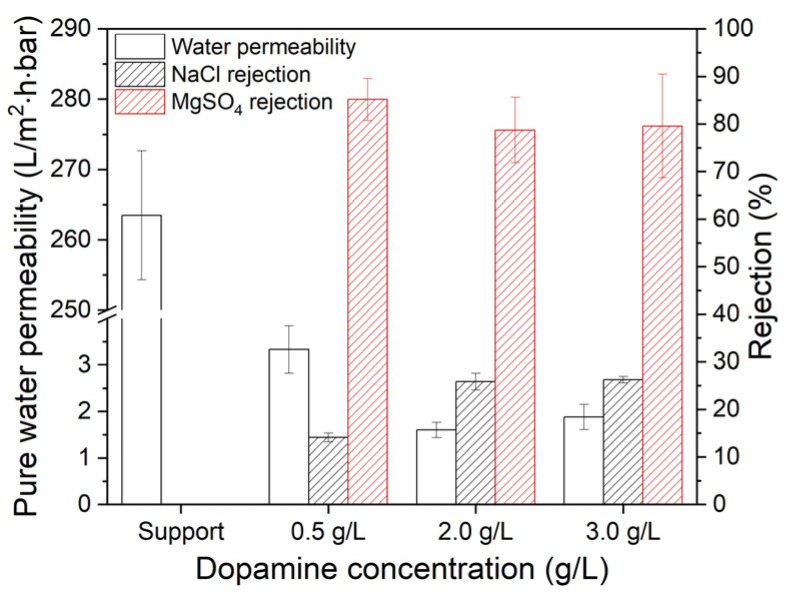
Effect of dopamine concentration (coating temperature: 25 °C) on the UPW permeability and rejection of NaCl and MgSO_4_. Rejection is measured with a salt concentration of 2 g/L at 5 bar. Average values and standard deviations represent at least four (UPW permeability) and two (rejection measurements) measurements, respectively.

UPW permeability and rejection measurements are presented in Figure 7.

The pristine PES membrane has a permeability of 264 ± 9 L/m^2^·h·bar and no salt retention. PDA coating on the PES support drastically decreases the permeability and increases the rejection due to pore coverage by the PDA layer. As can be seen in Figure 7, the permeability decreases with a factor 2 when the dopamine concentration increases from 0.5 g/L to 2.0 g/L as a result of the increased PDA layer thickness, due to the increased PDA deposition at higher dopamine concentrations without changing any further when using 3.0 g/L. Kasemset et al. [32] coated a polysulfone ultrafiltration membrane with PDA using varying dopamine concentrations (0–8 g/L) and observed similar behavior in water permeance. At low initial dopamine concentrations, permeance was higher which decreased gradually and while at concentrations from 2 g/L on, no significant further decrease was observed with increasing dopamine concentration. Our permeation data confirm the FESEM and XPS depth profiling observations. A trade-off is visible between the permeability and the NaCl rejection as more deposition of PDA and thicker PDA layers resulting in increased salt rejection. One of the effects on the salt rejection is the surface charge which influences the rejection due to charge exclusion. However, zeta potential measurements (Figure 6) show that the initial dopamine concentration and coating temperature have no significant influence on the zeta potential and isoelectric point of the membranes. Therefore, the salt rejection is mostly affected by the size exclusion. The NaCl rejection increases with dopamine concentration up to a value of 2.0 g/L. The MgSO_4_ rejection of the coated membranes is always higher than that for NaCl due to steric exclusion of MgSO_4_, which has a larger hydrodynamic radius (hydrated ionic radius of Mg^2+^: 0.395 nm; SO_4_^2−^: 0.300 nm; Na^+^: 0.360 nm; and Cl^−^: 0.270 nm [40]). However, the dopamine concentration does not affect the MgSO_4_ rejection. Independent of the coating concentration, the MgSO_4_ retentions remain stable at around 80%. In addition, the rejection of the MgSO_4_ is not dependent on the coating thickness. PDA coating thickness and molecular weight cut-off (MWCO) of PDA-coated polysulfone ultrafiltration membranes were also investigated by Kasemset et al. [32]. It was found that despite the increased thickness of the coating at increasing dopamine concentrations, the MWCO remained the constant. This explains the similar rejection values of the coatings with initial dopamine concentrations between 2.0 and 3.0 g/L. This suggests that rejection is affected by pore size more than the coating thickness.

##### Effect of Coating Temperature

**Figure 8:**
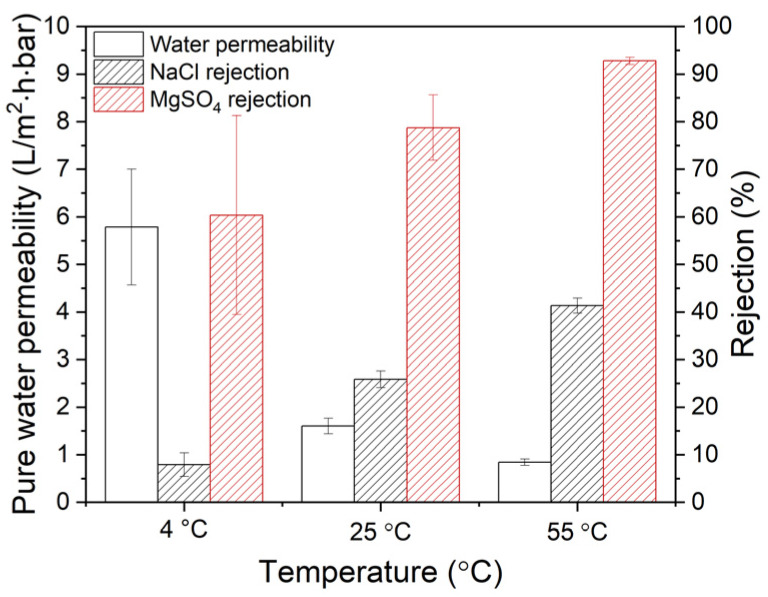
Effect of coating temperature (dopamine concentration: 2.0 g/L) on the UPW permeability and rejection of NaCl and MgSO_4_. Rejection is measured with a salt concentration of 2 g/L at 5 bar. Average values and standard deviations represent at least four (UPW permeability) and two (rejection measurements) measurements, respectively.

The effect of coating temperature on the UPW permeability and salt rejections can be seen in Figure 8.

An increase in the coating temperature decreases the permeability of the membranes significantly and increases both NaCl and MgSO_4_ rejections. PDA membranes coated at 4 °C show the highest water permeability and the lowest salt rejection. This is due to the thin PDA layer. Increasing PDA coating temperature decreased the permeability of the membranes and increased the retention of the salts. This is predominantly caused by two factors. First, the increased coating temperature accelerates the deposition of PDA and increases the thickness of the layer. The second factor is the formation of a denser PDA layer due to the smaller PDA aggregates. The membrane that was coated at 55 °C has the thickest PDA layer and thus shows the lowest permeability (0.84 L/m^2^·h·bar), which is 7 times lower compared to the 4 °C; meanwhile, the salt retention increases from 8 to 41% for NaCl and from 61 to 93% for MgSO_4_. These results show the strong effect of especially the coating temperature on the membrane separation performance while the effect of dopamine concentration is less.

#### 3.5.2. Forward Osmosis Performance

##### Effect of Dopamine Concentration

**Figure 9:**
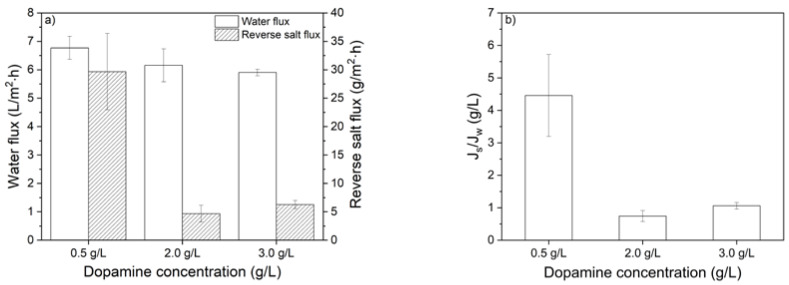
Effect of dopamine concentration on (**a**) water flux and reverse salt flux and (**b**) specific reverse salt flux (J_s_/J_w_) of the membranes investigated. Coating temperature: 25 °C. Feed solution: UPW. Draw solution: 1.2 M MgSO_4_. Co-current cross flow: 25 cm/s. Average values and standard deviations represent two replicate samples.

Subsequently, the FO membrane performance is determined and characterized in terms of water flux, reverse salt flux and specific reverse solute flux (J_s_/J_w_). Figure 9a shows the water flux and reverse salt flux of the membranes that are coated with different dopamine concentrations at 25 °C.

The water flux of the membranes did not change significantly with increasing dopamine concentration, which is in contrast to the observations from the permeability measurements (Figure 7). On the other hand, the reverse salt flux decreases by 75% when using a coating containing 2.0 or 3.0 g/L initial dopamine concentration without compromising the water flux. This behavior clearly highly favors the specific reverse salt flux, which decreased from 4.5 to ~1 g/L, significantly improving the FO performance by maintaining the water flux but lowering the reverse salt flux (Figure 9b). Although the coating layers thickness is increased with higher coating concentrations, a decline in water flux is not observed. This is attributed to the corresponding higher salt rejecting due to the thicker coating layer, thus decreasing the reverse salt flux, which eventually prevents the loss in effective osmotic pressure during the FO operation and reduces internal concentration polarization. Membranes that are coated with 2.0 and 3.0 g/L dopamine concentrations have an equal J_s_/J_w_ ratio as a result of similar water flux and reverse salt flux values. These are significantly better when compared to the membranes coated with 0.5 g/L dopamine that shows especially higher reverse salt fluxes at similar water flux values.

##### Effect of Coating Temperature

**Figure 10:**
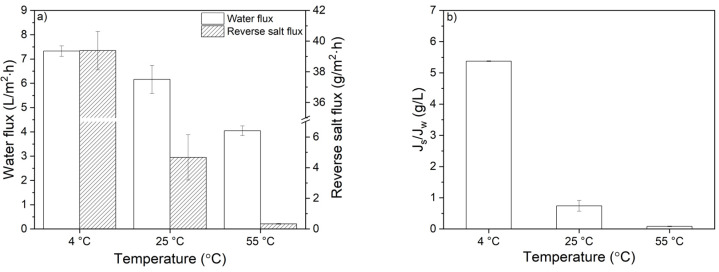
Effect of temperature on (**a**) water flux and reverse salt flux and (**b**) specific reverse salt flux (J_s_/J_w_) of the membranes investigated. Dopamine concentration: 2.0 g/L. Feed solution: UPW. Draw solution: 1.2 M MgSO_4_. Co-current cross flow: 25 cm/s. Average values and standard deviations represent two replicate samples.

The effect of temperature on water and reverse salt flux is depicted in Figure 10a.

A small decline in water flux and a very steep decrease in reverse salt flux were observed when higher coating temperatures were used. Due to the slow reaction kinetics and low PDA deposition rates, PDA coatings applied at 4 °C resulted in high water fluxes as well as in extremely high reverse salt fluxes. The lowest reverse salt flux (0.34 g/m^2^·h) is achieved at the highest coating temperature, i.e., 55 °C, which is 14 times lower than the reverse salt flux of the coating applied at room temperature and even 116 times lower than the coating applied at 4 °C. The water flux of these membranes decreases by only a factor of 1.2 and 1.8, respectively. The benefits of the greatly reduced reverse salt flux and the hardly decreased water flux are the reduced salt leakage from draw solution towards feed solution during the product concentration, thereby preventing the decrease of the effective osmotic pressure. However, more importantly, the low reverse salt fluxes guarantee a high product purity and quality. The J_s_/J_w_ value of the membranes is also depicted in Figure 10b. As expected, the high salt permeation of the PDA membranes coated at 4 °C increases the J_s_/J_w_ ratio. On the other hand, the PDA membrane coated at 55 °C has the lowest ratio (0.08 g/L) giving the most favorable performance.

##### Performance Comparison with Commercial FO Membranes

The FO performance of the PDA-coated membranes is compared with the FO performance of the commercial cellulose triacetate (CTA) and thin film composite (TFC) FO membranes. Comparison is done with the values found from other studies which used MgSO_4_ as draw solution. For this purpose, PDA membranes coated at 55 °C are used since the best J_s_/J_w_ ratio is achieved with these membranes. Table 2 shows the water and reverse salt flux and J_s_/J_w_ ratio of the PDA-coated membranes at 55 °C, commercial CTA, and TFC membranes.

Achilli et al. [2] investigated the FO performance of commercial CTA FO membrane using different draw solutions. The MgSO_4_ concentration of the draw solution used is 1.17 M, similar to the MgSO_4_ concentration used in our study. Their findings show that the CTA membrane has a water flux of 5.54 L/m^2^·h and a reverse salt flux of 1.2 g/m^2^·h resulting in a J_s_/J_w_ ratio of 0.21 g/L. Phuntsho et al. [41] also studied the effect of different draw solutions and concentrations on the water and reverse salt fluxes and the J_s_/J_w_ ratio for commercial CTA FO, TFC FO, and TFC RO membranes. Table 2 only reports the values of the CTA FO membrane, as only for that membrane MgSO_4_ was used as draw solution. Water and reverse salt flux values of this membrane coincide with the values obtained by Achilli et al. [2], showing a similar J_s_/J_w_ ratio of ~0.33 g/L. The PDA-coated membranes of our study and the CTA membranes have very similar water flux values but the reverse salt flux of these custom-made PDA-coated membranes is 10 times smaller though. Consequently, the specific reverse salt flux, J_s_/J_w_, of this membrane is also 10 times lower, resulting in lower salt intrusion and a higher quality concentrated product streams for the newly developed PDA-coated PES membranes. Next to improved product quality, a low specific reverse salt flux also ends up in higher revenues due to lower process costs, less draw solution consumption, lower regeneration costs, and less deterioration of the product resulting in a higher price. TFC FO membranes on the other hand have higher water flux and lower reverse salt flux values compared to the PDA-coated PES membranes resulting in 4 times lower J_s_/J_w_ ratio, but at a much lower MgSO_4_ solution concentration (0.9 M) [42]. In that perspective, the reverse salt flux of the PDA-coated PES membranes thus clearly competes with the TFC FO membrane. The higher water flux of the TFC FO membrane is the result of the much more open support structure of this membrane giving rise to significantly less internal concentration polarization. The PES UF support that is used in our study for PDA coating is chosen for its constant quality and optimal structure for defect free PDA coating, but it is not optimized for FO.

## 4. Conclusions

In this study, PDA is successfully coated on porous PES UF membranes. The effect of dopamine concentration and coating temperature was investigated. Both increased dopamine concentration and higher coating temperatures show improvements in especially lowering the reversed salt flux of the membranes. This is due to increased thickness of the PDA layer and/or a more dense structure which is induced by accelerated PDA deposition on the surface as well as formation of the finer aggregates as revealed by FESEM and XPS measurements. In the dead-end filtration, best performance is obtained by using a coating solution containing 2.0 g/L dopamine at 55 °C. This gives membranes with the lowest permeability (0.84 L/m^2^·h·bar) and the highest NaCl (41%) and MgSO_4_ (93%) salt rejections. These membranes also show a 114 times lower reverse salt flux and a 1.8 times lower water flux in FO operation compared to membranes with coatings applied at 4 °C. Moreover, this is due to the thicker PDA layer and denser structure as a result of accelerated deposition and the formation of smaller aggregates at high temperature. As a result, a low J_s_/J_w_ ratio is obtained (0.08 g/L) which is a necessity for FO applications to obtain a high product quality with low salt contamination. The low J_s_/J_w_ ratio of the PDA membrane coated at 55 °C also outperforms commercial CTA FO membranes in terms of high product quality due to the drastically low reverse salt flux and reasonable water flux values. PDA membrane coated at 55 °C has promising flux values that can be comparable to TFC FO membrane despite the adverse effects of the PES support structure.

## Figures and Tables

**Table 1:** Surface compositions in atomic percentages of the pristine PES and PDA-coated PES membranes.

Dopamine Concentration (g/L)	Temperature (°C)	C 1s (%)	N 1s (%)	S 2p (%)	N/C	N/S
Pristine PES	-	74.99	2.02	6.23	0.03	0.32
0.5	25	72.71	8.15	0.13	0.11	62.69
2.0	25	72.55	7.34	0.05	0.10	146.80
3.0	25	72.19	7.43	0.08	0.10	92.88
2.0	4	72.98	8.11	0.86	0.11	9.43
2.0	55	71.45	8.63	0.07	0.12	123.29

**Table 2:** Water flux, reverse salt flux, and J_s_/J_w_ ratio of the PDA membranes coated at 55 °C, CTA, and TFC commercial FO membranes.

Membrane	Water Flux (L/m^2^·h)	Reverse Salt Flux (g/m^2^·h)	J_s_/J_w_ Ratio (g/L)	Reference
PDA coated at 55 °C	4.05 ± 0.20	0.34 ± 0.03	0.08 ± 0.01	This work
CTA FO	5.54	1.20	0.21	[2]
CTA FO	~ 4.33	~ 1.50	~ 0.33	[41]
TFC FO	~ 7.00	~ 0.13	~ 0.02	[42]
